# Supplementary material for: A unified framework for species spatial patterns: Linking the occupancy area curve, Taylor's Law, the neighborhood density function and two‐plot species turnover
Source: Ecol Lett. 2021 Aug 4;24(10):2043–53. doi: 10.1111/ele.13788 (PMC8518128; doi:10.1111/ele.13788)
Supplement: Supplementary file 4 — Supplementary Material [file ELE-24-2043-s004.zip › calcs/6-figures/spp-preds/psycli_preds.pdf]

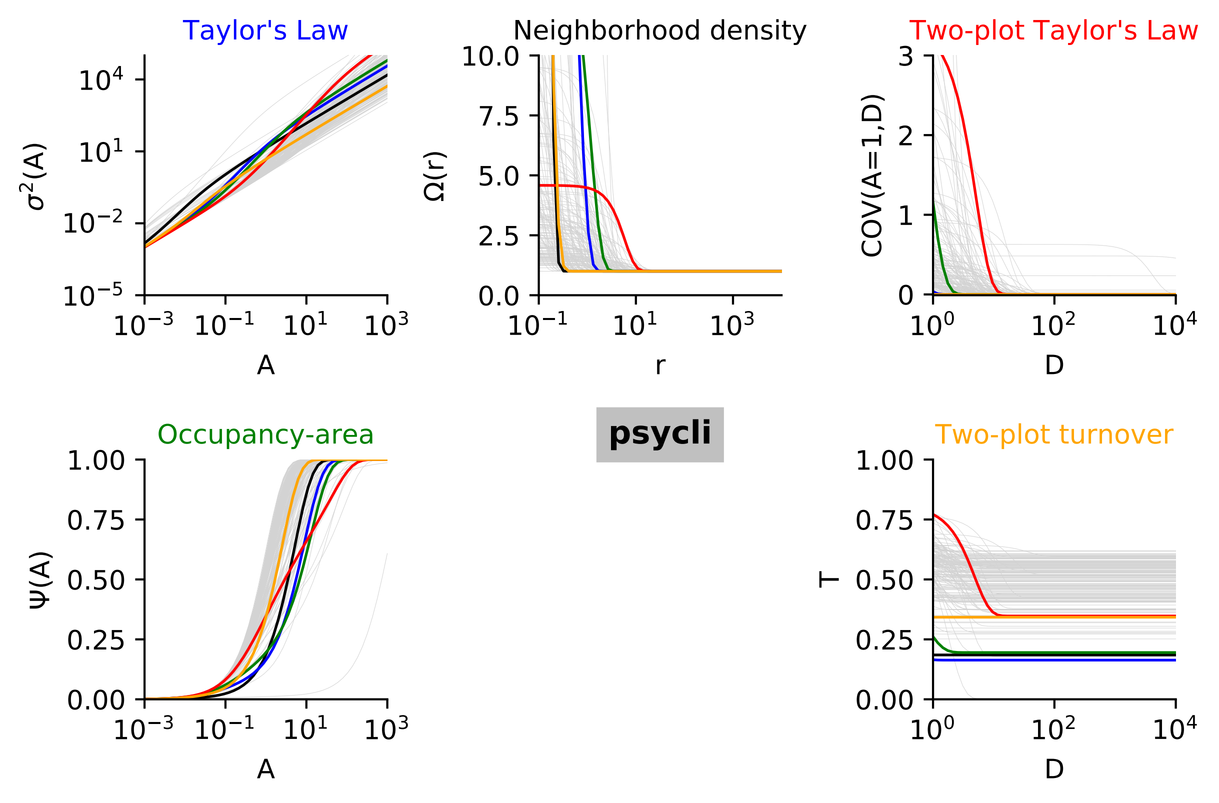

Taylor's Law
Neighborhood density
Two-plot Taylor's Law
$\sigma^2(A)$
$\Omega(r)$
COV(A=1,D)
A
r
D
Occupancy-area
psycli
Two-plot turnover
$\Psi(A)$
T
A
D
